# Mutual Facilitation Among Invading Nuttall’s Waterweed and Quagga Mussels

**DOI:** 10.3389/fpls.2019.00789

**Published:** 2019-06-26

**Authors:** Benjamin Wegner, Anna Lena Kronsbein, Mikael Gillefalk, Klaus van de Weyer, Jan Köhler, Elisabeth Funke, Michael T. Monaghan, Sabine Hilt

**Affiliations:** ^1^Department of Ecosystem Research, Leibniz-Institute of Freshwater Ecology and Inland Fisheries (IGB), Berlin, Germany; ^2^Faculty VI: Planning, Building and Environment, Institute for Ecology, Technical University Berlin, Berlin, Germany; ^3^Lanaplan GbR, Nettetal, Germany

**Keywords:** macrophyte, lake, invasional meltdown hypothesis, competition, invasive species

## Abstract

Nuttall’s waterweed (*Elodea nuttallii*) is the most abundant invasive aquatic plant species in several European countries. *Elodea* populations often follow a boom-bust cycle, but the causes and consequences of this dynamics are yet unknown. We hypothesize that both boom and bust periods can be affected by dreissenid mussel invasions. While mutual facilitations between these invaders could explain their rapid parallel expansion, subsequent competition for space might occur. To test this hypothesis, we use data on temporal changes in the water quality and the abundance of *E. nuttallii* and the quagga mussel *Dreissena rostriformis bugensis* in a temperate shallow lake. Lake Müggelsee (Germany) was turbid and devoid of submerged macrophytes for 20 years (1970–1989), but re-colonization with macrophytes started in 1990 upon reductions in nutrient loading. We mapped macrophyte abundance from 1999 and mussel abundance from 2011 onwards. *E. nuttallii* was first detected in 2011, spread rapidly, and was the most abundant macrophyte species by 2017. Native macrophyte species were not replaced, but spread more slowly, resulting in an overall increase in macrophyte coverage to 25% of the lake surface. The increased abundance of *E. nuttallii* was paralleled by increasing water clarity and decreasing total phosphorus concentrations in the water. These changes were attributed to a rapid invasion by quagga mussels in 2012. In 2017, they covered about one-third of the lake area, with mean abundances of 3,600 mussels m^−2^, filtering up to twice the lake’s volume every day. The increasing light availability in deeper littoral areas supported the rapid spread of waterweed, while in turn waterweed provided surface for mussel colonization. Quantities of dreissenid mussels and *E. nuttallii* measured at 24 locations were significantly correlated in 2016, and yearly means of *E. nuttallii* quantities increased with increasing mean dreissenid mussel quantities between 2011 and 2018. In 2018, both *E. nuttallii* and dreissenid abundances declined. These data imply that invasive waterweed and quagga mussels initially facilitated their establishment, supporting the invasional meltdown hypothesis, while subsequently competition for space may have occurred. Such temporal changes in invasive species interaction might contribute to the boom-bust dynamics that have been observed in *Elodea* populations.

## Introduction

Positive interactions can play a decisive role in shaping communities and regulating ecosystem structure and function (Halpern et al., 2007; Brooker et al., 2008; Soliveres et al., 2015). Based on the widespread occurrence of positive interactions between non-native species, Simberloff and Von Holle (1999) developed the “invasional meltdown hypothesis (IMH)” suggesting that non-indigenous species can facilitate one another’s invasion in various ways, increasing the likelihood of survival and/or of ecological impact, and possibly the magnitude of impact. A review of invasions in the Great Lakes by Ricciardi (2001) indeed showed that direct positive (mutualistic and commensal) interactions among introduced species were more common than negative interactions. Recently, Braga et al. (2018) used the hierarchy-of-hypotheses approach to differentiate key aspects of the IMH. While the majority of studies supported the IMH, studies at the community or ecosystem level were rare, especially for cases where two non-native species interact and both species are affected. There are also only few studies on indirect positive interactions, as these are often difficult to detect and measure, and thus often neglected (White et al., 2006).

**Figure 1:**
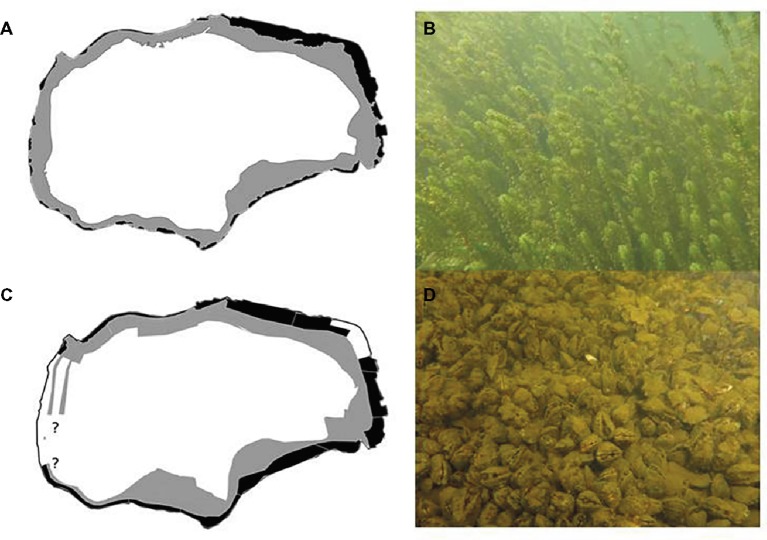
Colonized area of submerged macrophytes **(A)** in 1999 (black area) and 2017 (black and grey area) and of dreissenid mussels in 2017 **(C)** in Lake Müggelsee. Black area in **(C)**: hypothetical distribution of *Dreissena polymorpha* before *D. r. bugensis* invasion down to 2 m due to restriction to hard substrates, black area + grey area in **(C)**: combined distribution of both dreissenid species in 2017. ?: uncertainties in the dreissenid distribution in 2017 due to restricted accessibility *via* motorboat. Photos: *Elodea nuttallii* **(B)** and *D. r. bugensis* **(D)** in Lake Müggelsee in 2017.

Here, we present an observational field study on the rapid invasion of two non-indigenous species and their potential direct and indirect interactions during this process in a temperate polymictic freshwater lake. Similar to many other freshwater bodies, Lake Müggelsee (Germany) had almost completely lost its submerged macrophytes due to eutrophication during the last century. Following significant reductions in nutrient loading, macrophytes slowly started re-colonizing the lake, but deeper littoral areas still remained sparsely covered or devoid of macrophytes (Hilt et al., 2013, 2018). During the last decade, two major invaders entered the lake, Nuttall’s waterweed (*Elodea nuttallii*, Figure 1B) and the quagga mussel (*Dreissena rostriformis bugensis*, Figure 1D), and were both first detected around 2011/12. We hypothesize that mutual facilitation among these invaders could explain their rapid parallel expansion.

*E. nuttallii,* native in Northern America, was introduced into Europe in 1939. It spread rapidly and became the most abundant non-indigenous aquatic plant species in several countries, owing to its ability to easily colonize new areas by fragments (Wolff, 1980; Hussner, 2012). It is also a common pioneer species colonizing lakes after restoration measures such as biomanipulation of the fish community (Hilt et al., 2018). Excessive growth can cause severe nuisance for fishery and tourism in lakes (Hilt et al., 2006; Zehnsdorf et al., 2015) and can decrease biodiversity by out-competing less robust native macrophyte species and by releasing allelochemicals (Barrat-Segretain, 2005; Kelly and Hawes, 2005; Erhard and Gross, 2006). *Elodea* species often follow a boom-bust dynamic (Simberloff and Gibbons, 2004; Strayer et al., 2017); however, the causes and consequences of these dynamics are yet unknown. In principle, *E. nuttallii* could facilitate an invasion of *D. r. bugensis* directly by providing surface for attachment of mussels and indirectly by oxygen production. Survival of quagga mussels is strongly reduced at low oxygen concentrations (De Ventura et al., 2016). While native macrophytes also provide surface and oxygen, invasive macrophytes are often faster in colonizing newly available habitats after disturbances (Chytry et al., 2008) and *E. nuttallii* is expected to spread fast into deeper littoral areas that have begun to receive sufficient light for macrophyte growth after a quagga mussel invasion. Invasive macrophytes have also been found to specifically facilitate the establishment of invasive bivalves (Michelan et al., 2014), but the mechanisms remain unknown. In contrast, dense macrophytes can have negative effects on phytoplankton abundance (Scheffer et al., 1993) and thus may restrict food availability for mussels (Reusch and Williams, 1999).

*D. r. bugensis*, native in the Ponto-Caspian region, was first detected in Western Europe in 2004 (Paulus et al., 2014). It is one of the world’s most problematic biological invaders and has been shown to affect the biogeochemistry, flora, and fauna of lakes and rivers across North America and Eurasia. It can shift aquatic food webs and energy flow from pelagic-profundal to benthic-littoral energy pathways (Higgins and Vander Zanden, 2010). *D. r. bugensis* could indirectly facilitate the invasion of *E. nuttallii* by increasing the light availability at the littoral of lakes due to its high filtration capacity. Direct effects of dreissenid mussels on submerged macrophytes through nutrient relocation into littoral areas seem possible, but appeared to be less important than the positive effects associated with increased light penetration (Zhu et al., 2007).

We tested the hypothesis of mutual facilitation between these two non-native species using data from Lake Müggelsee on the coverage and quantity of submerged macrophytes from 1999–2018 and of *E. nuttallii* and *D. r. bugensis* during their establishment from 2011–2018, and water quality parameters such as water transparency, concentrations of total phosphorus (TP) and chlorophyll *a.*

## Materials and Methods

### Lake Müggelsee

Lake Müggelsee is the largest lake in Berlin (Germany, 52°26′, 13°39′, Figure 1A). It has a surface area of ~7.4 km^2^, a volume of 36,560,000 m^3^, a water retention time of around 100 days (Gillefalk et al., 2018), and a catchment area of 7,000 km^2^. It is a shallow, polymictic lake with a mean depth of 4.9 m and maximum depth of about 8 m (Hilt et al., 2013) with extensive shallow areas at the northeastern, eastern, and southeastern shores. In 1970, the lake had almost completely lost its submerged macrophytes as a result of eutrophication. The external loading of nitrogen (N) and phosphorus (P) to Lake Müggelsee decreased significantly between the late 1970s and 2016. Total N (TN) loads decreased from 140 ± 38 g TN m^−2^ a^−1^ in the 1980s to 30 ± 11 g TN m^−2^ a^−1^ in the decade 2007–2016. Total P (TP) loads decreased from 6 ± 1 to 2 ± 0.4 g TP m^−2^ a^−1^ in the same period (Shatwell and Köhler, 2019). Macrophytes started re-colonizing the lake from 1990 onwards along with reductions in external nutrient loading. Re-colonization, however, was slow and the macrophyte community was dominated by a few species, mainly sago pondweed [*Stuckenia pectinata* (L.) Börner] for about 20 years (Hilt et al., 2013, 2018).

From 1978 onwards, water samples have been taken and Secchi disk transparency in the water column recorded weekly (summer) or biweekly (winter). Volumetrically weighted integrated samples were taken from 21 subsamples from five different points on the lake since 1987 and concentrations of total phosphorus (TP) and chlorophyll *a* (chl *a*) have been determined [for details see Shatwell and Köhler (2019)]. We have used data for the last 20 years (1999–2018).

### Submerged Macrophytes

We used two different approaches for macrophyte mapping: (1) a transect-based mapping which allowed us to follow the detailed development of species, their abundances in different depth zones, and maximum colonization depth (MCD) between 1999 and 2018, and (2) an entire lake mapping of the abundance of macrophytes and maximum colonization depth (MCD) of macrophytes to estimate the total lake coverage in 1999 and 2017.

Transect data are available for 8 years, and macrophytes were always mapped in June or early July. In 2006, 2011, and 2014–2018, submerged macrophytes were mapped at eight transects covering the diversity of habitats in the lake (in 2006 only 5 of those) according to the PHYLIB method developed for the implementation of the EU Water Framework Directive in Germany (Schaumburg et al., 2004). In addition, data of a detailed mapping in 1999 (Körner, 2001) were transformed into the PHYLIB method for the eight selected transects, which was possible due to the low abundance and low MCD. In 1999 and 2006, mappings were performed using wading and aquascopes because MCD (determined by aquascopes and extensive raking of deeper areas from a boat) were low, while scuba diving has been used since 2011.

Maximum colonization depths were recorded for each transect, and abundances were estimated in up to four depth zones (0–1, 1–2, 2–4, and 4–6 m) based on a five-degree scale (1: very rare; 2: rare; 3: common; 4: frequent; and 5: abundant). To obtain at macrophyte quantities, abundance data were exponentiated with 3 to reflect the three-dimensional development of macrophytes (Schaumburg et al., 2004).

Entire lake mappings of macrophytes were performed in 1999 by wading with an aquascope and GPS (for details see Körner, 2001) and in 2017 by using an underwater camera (see Van de Weyer et al., 2007).

### Abundance and Quantities of Dreissenid Mussels

**Figure 2:**
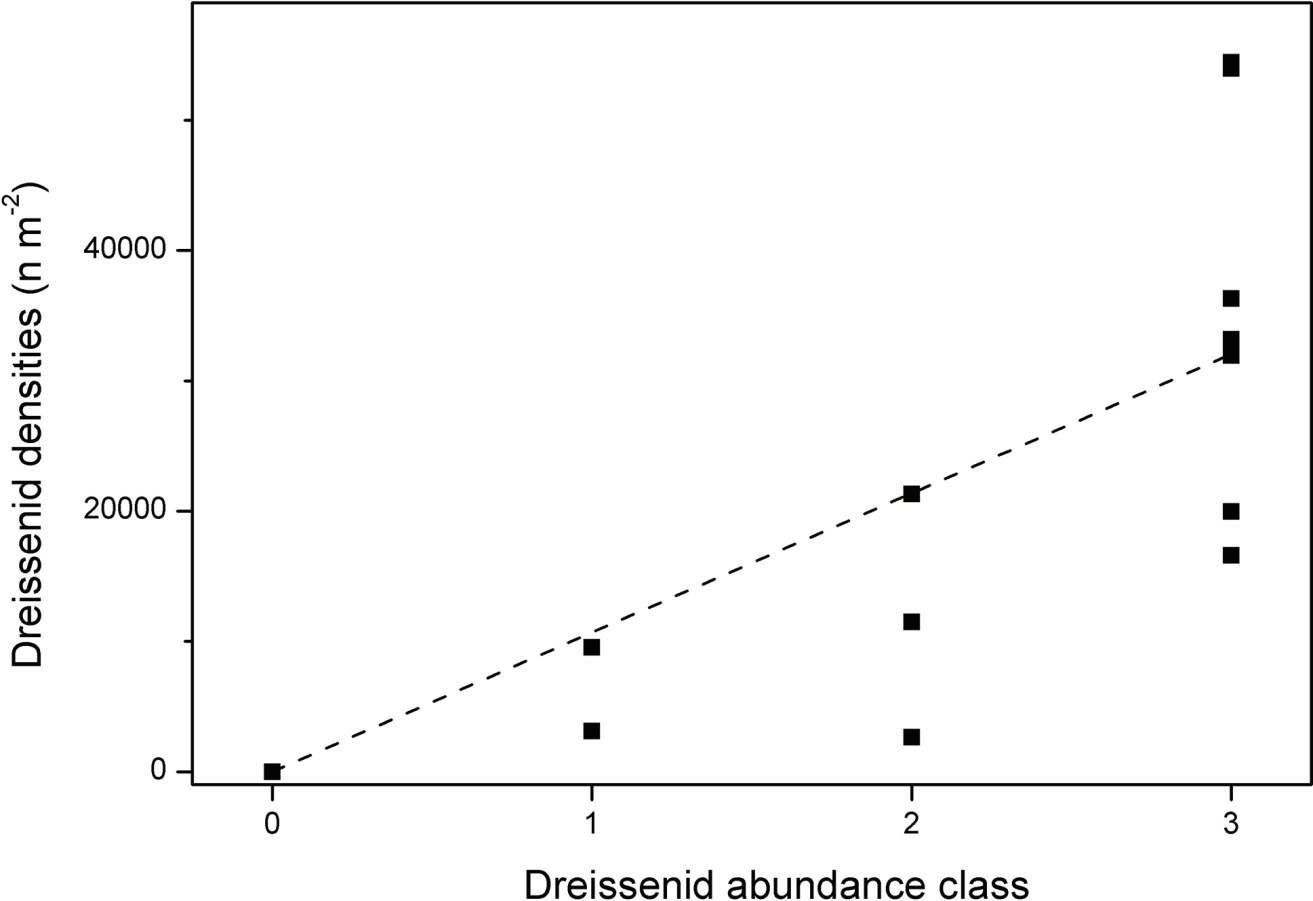
Dreissenid mussel densities per abundance class at three transects in Lake Müggelsee in 2017.

Data on dreissenid mussel abundance are only available since 2011. We also used two different approaches for dreissenid mussel mapping: (1) a transect-based mapping which followed their abundances in different depth zones (same as for macrophytes, see section “Submerged Macrophytes“) in 2011 and 2015–2018, and (2) an entire lake mapping of the dreissenid mussel abundance and their maximum colonization depth, used to estimate the total lake coverage and to calculate filtration activities in 2017 (see section “Determination of Filtration Rates by Dreissenids”). For the transect-based mapping, two approaches were applied. In 2011, 24 sediment samples were taken at different water depths using a Van Veen grab (sampling 600 cm^2^) and mussels were counted at eight transects in parallel to the macrophyte mapping. In 2015–2018, the abundance of dreissenid mussels was recorded by scuba divers in parallel to macrophyte mapping at eight transects using four abundance classes: 0: no dreissenids, 1: up to 33% sediment coverage, 2: 33–66%, and 3: 66–100% sediment coverage. To transform mussel numbers from 2011 into these abundance classes, divers collected dreissenid mussels at 13 locations with different depths and abundance classes at three transects from an area of 170 cm^2^ in 2017. A significant linear regression between mussel numbers and abundance class (*y* = 10,680*x*, *R*^2^ = 0.52, *p* = 0.003; Figure 2) allowed us to calculate threshold levels for the transformation of mussel numbers in 2011 into abundance classes (0: no mussels, 1: 1–10,680 dreissenid mussels m^−2^, 2: 10,681–21,360 dreissenid mussels m^−2^, 3: >21,361 dreissenid mussels m^−2^). Mean quantities were calculated by averaging (square-root) dreissenid abundances measured at the respective transects in different depths inside of each depth class.

### Determination of Dreissenid Species and Densities

To determine densities and species distributions of dreissenids, 40 samples were taken from different water depths at 13 transects (3–5 samples per transect) and along the northern shore littoral between October 2017 and January 2018 using an Ekman-Binge sediment sampler (Hydro-Bios®) with a sample area of 225 cm^2^. Locations with dreissenid coverages of 66–100% were selected to take sediment samples at water depth intervals of 1 m (±0.5 m). Five locations with dense dreissenid coverage on rock fill or shallow sandy parts were sampled with a shovel. Samples were sieved (0.5 mm) and stored in 1 L wide neck plastic containers at 5°C until further treatment (maximum 20 days). Samples were separated into living and dead *D. r. bugensis* and *Dreissena polymorpha*, respectively, based on morphological identification and each fraction was counted. Living individuals were measured and assigned to size-classes of <1.0, 1.0–2.0, and >2.0 cm.

### Genetic Verification of Differentiation Between Dreissenid Species

Species determination of dreissenids based on morphological characteristics has been proven difficult or inconsistent due to habitat-specific growth patterns (May and Marsden, 1992; Beggel et al., 2015). Beggel et al. (2015) suggested applying molecular methods to differentiate between *D. r. bugensis* and *D. polymorpha*, and we therefore sequenced the standard mitochondrial DNA Cytochrome c oxidase I (*cox1*) barcode sequence to verify our morphological identifications (Marescaux and Van Doninck, 2013). Total genomic DNA was extracted from 42 *Dreissena* individuals sampled in 2017 of one eastern, one western, and one southwestern location in depths of 2.20, 2.30, and 2.50 m, respectively, using the Dneasy Blood and Tissue kit (Qiagen, Hilden, Germany) according to the manufacturer’s guidelines. A 658-bp fragment of *cox1* was amplified following Marescaux and Van Doninck (2013) with the following modifications. Amplifications were performed in a total volume of 25 μl (with 1 μl of DNA, 1× mi-Taq only reaction buffer (green cap; Metabion, Planegg/Steinkirchen, Germany), 200 μM of dNTPs (Metabion), 0.4 μM of each primer, and 1 U of mi-Taq only DNA polymerase (Metabion). PCR cycling conditions were 95°C for 4 min, 30 cycles of 95°C for 45 s, 49°C for 45 s and 72°C for 45 s, and 72°C for 10 min. Sanger sequencing in both directions of COI fragments was performed by LGC Genomics GmbH (Berlin, Germany). Sequences were merged and quality-trimmed using Geneious 10.0.6 (Biomatters ApS, Aarhus, Denmark) and then sequences were used as queries in searches of the NCBI nucleotide database using the *blastn* alorithm^1^.

Comparison with NCBI database indicated that our morphological identification was correct in each case. There were >100 bp changes between the two species, and no differences among individuals sampled from Lake Müggelsee. Our *D. r. bugensis* haplotype matched that of individuals from the Meuse River in eastern France, and the *D. polymorpha* haplotype was an exact match to individuals sampled from Poland and North America (New York, Ontario).

### Determination of Filtration Rates by Dreissenids

In November 2017, dreissenid coverage of the sediments was mapped in the entire lake using an underwater camera mounted on a motorboat. A SELVAG® OC-1 outdoor surveillance camera attached to an 8-m long monitor microphone TS cable was directed toward the lake bottom. The analogue camera image was converted to a digital signal using a USB-audio and video converter and displayed on a computer screen. Dreissenid mussel sediment coverages were assessed while looking at the live camera image and assigned to the four coverage classes (see section “Abundance and Quantities of Dreissenid Mussels”) simultaneously with coordinates, water depth, date, and time. To map areas deeper than 5 m, where visibility was usually lower, the boat was anchored at depth-intervals of 0.5 m to enable a steady image of the lake bottom.

We subdivided the whole lake into six radial sub-areas (in terms of cardinal directions) while ensuring that a minimum of four samples (see section “Determination of dreissenid species and densities”) was located in each sub-area. Median population densities of dreissenids were determined for each sub-area. Population densities were transformed (square-root) to achieve normal distribution, and the effect of sub-area on population densities was analyzed *via* one-way ANOVA. A subsequent *post-hoc* unequal n Tukey’s HSD-test was used to determine significant differences among sub-areas regarding the mussels’ median population densities. Sub-areas with representative median population densities were then intersected with the coverage classes of dreissenids to yield dreissenid abundance for each lake-sub-area.

Since samples were generally retrieved from locations with dreissenid sediment coverages of 3 (66–100%), population densities of sub-areas with sediment coverages of 2 (33–66%) were multiplied with the correction factor 0.6 and those of sediment coverages classes 1 (1–33%) with factor 0.3. By this means, dreissenid abundances per lake-area were calculated and ultimately summated to a whole-lake abundance of each species, as well as both species combined. For the calculation of the filtration capacity of all dreissenids, we used filtration rates (FR) determined for *D. polymorpha* in Reeders et al. (1989) in two Dutch shallow lakes (Ijsselmeer and Markermeer). Reeders and Bij de Vaate (1990) found that the effect of season and temperature only set a wide limit to the filtration activity, but water temperature below 5°C significantly decreased FR. Hence, FR were adjusted by duration (weeks) of water temperature of Lake Müggelsee with values >5°C (79%) and ≤ 5°C (21%) in 2017. Summed, seasonally adjusted FR were multiplied by median total abundance of both dreissenid species combined. To arrive at the fraction of lake water volume filtered per day, the product of total filtration per day was divided by the water volume of Lake Müggelsee. Mei et al. (2016) legitimized the application of FR reported for zebra mussels to quagga mussels.

### Statistics

We tested for significant correlations between the quantities of *E. nuttallii* and native macrophytes measured at 24 sites over 6 years (*n* = 144) using Spearman correlations. We also tested for significant correlations between the quantity of *E. nuttallii* and native macrophytes in the depth zone 2–4 m with MCD of macrophytes.

Relationships between the quantities of dreissenids and *E. nuttallii* or native macrophytes were investigated with Spearman correlations using the data available for eight transects and three depth zones (*n* = 24) separately for each of the years 2011 and 2015–18. Additionally, we tested for significant relationships between the yearly mean quantities of dreissenid mussels and *E. nuttallii* or native macrophytes for the period 2011–18 (*n* = 5) by best fit non-linear regressions as well as between dreissenid mussels and Secchi disc transparency in winter (October to March) and spring (April to June) by linear regressions. All statistical analyses were performed using SPSS 19.

## Results

### Submerged Macrophytes

**Figure 3:**
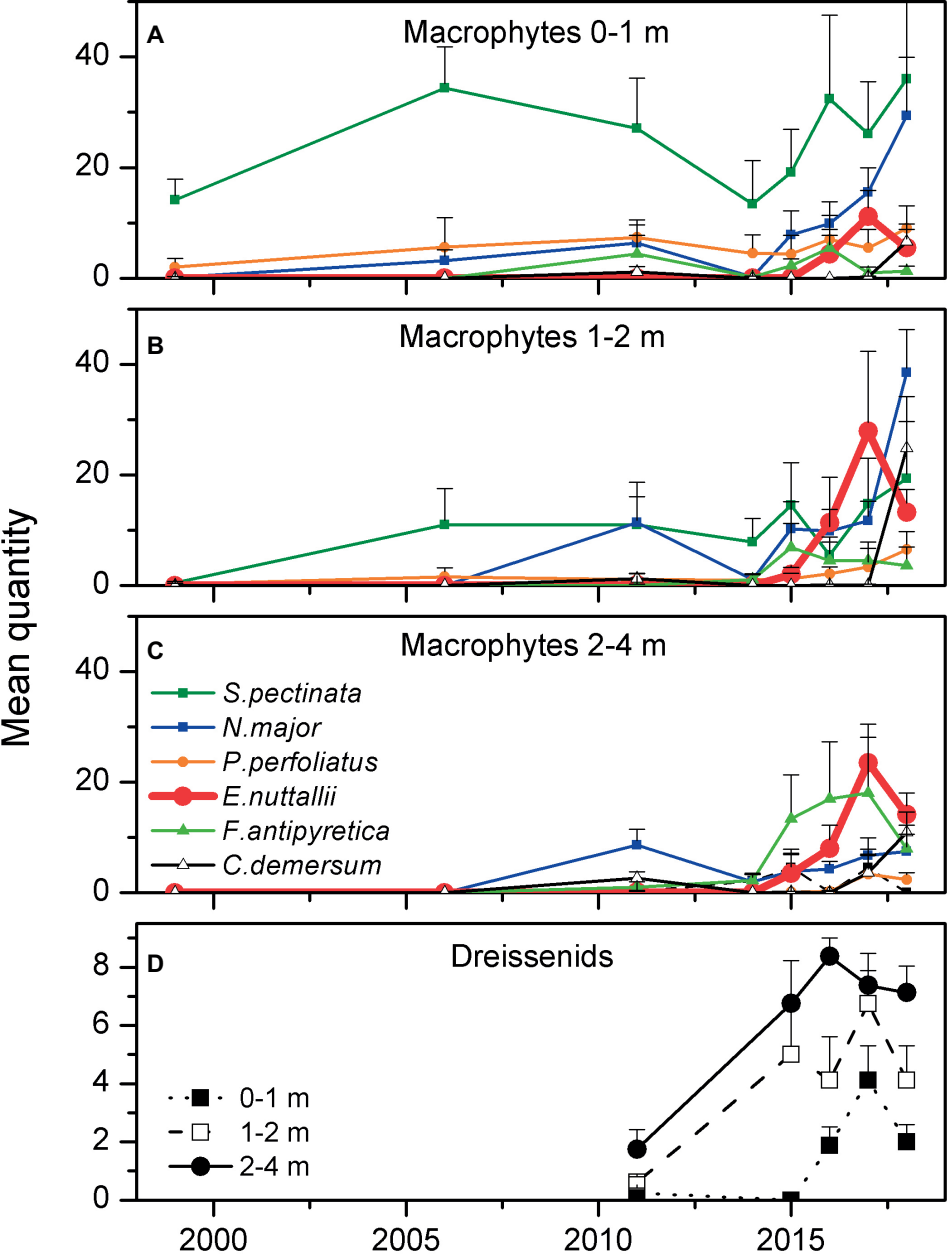
Mean quantity (see section “Submerged Macrophytes”) of the six most common submerged macrophyte species (+standard error) measured at eight transects in three different depth zones (**A**: 0–1 m, **B**: 1–2 m, **C**: 2–4 m) and mean quantity of dreissenid mussels (+standard error) in the different depth zones **(D)** at eight transects in Lake Müggelsee between 1999 and 2018.

**Figure 4:**
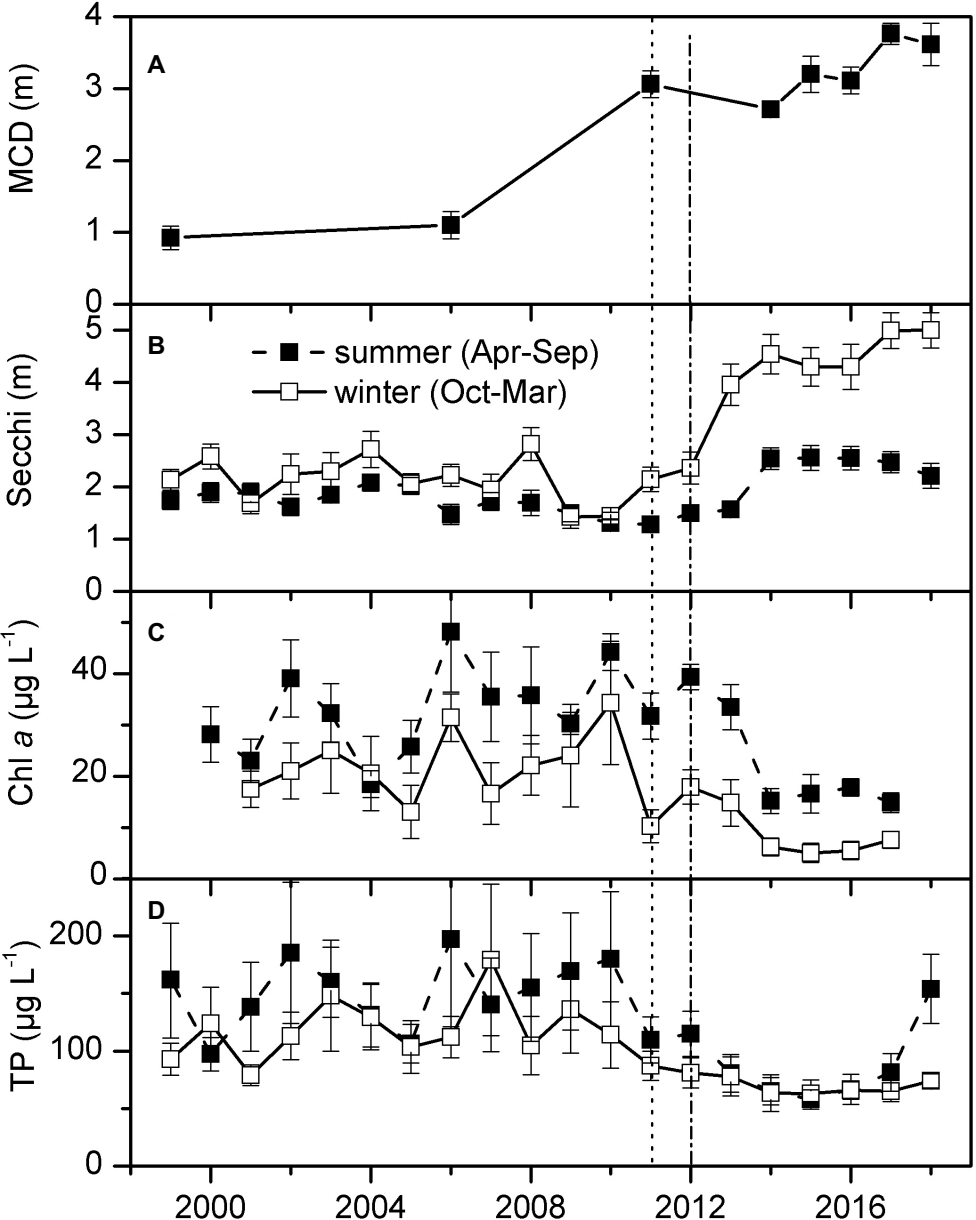
Maximum colonization depth of submerged macrophytes (MCD, measured at the end of June at 8 transects) **(A)**, summer (April–September) and winter (October–March) Secchi disk transparency **(B)** and concentrations of total phosphorus (TP) **(D)** and chlorophyll (chl) *a* in Lake Müggelsee [means of weekly (summer) or biweekly (winter) measurements ± standard error] **(C)**. Vertical lines represent first detection of *E. nuttallii* (dots) and assumed start of invasion of *D. r. bugensis* (dash-dot).

Total coverage of submerged macrophytes in Lake Müggelsee increased from 5% in 1999 to 25% in 2017 (Figures 1A,C). The six most abundant submerged macrophytes in Lake Müggelsee during its re-colonization were the native species *S. pectinata, Potamogeton perfoliatus, Najas major, Fontinalis antipyretica*, *Ceratophyllum demersum*, and (from 2011) the invasive *E. nuttallii*. Until 2006, only *S. pectinata, P. perfoliatus,* and *N. major* were present in significant quantities and hardly any macrophytes were found in depth zones below 2 m (Figures 3A–C). High mean quantities were only reached by *S. pectinata* in the shallowest depth zone (0–1 m). *E. nuttallii* was first discovered with low quantities at two transects at the south-eastern shore in depth zones 1–2 and 2–4 m in 2011. Subsequently, it spread to the entire lake and was present at all eight transects in 2017. Its quantity per site increased between 2011 and 2017 along with that of other native macrophyte species, and their quantities at the investigated sites were significantly positively correlated (Spearman correlation, *R* = 0.445, *p* < 0.001, *n* = 144). The number of native macrophyte species per site also increased during this period and was positively correlated to the quantities of native macrophytes and *E. nuttallii* (Spearman correlations, *R* = 0.691, *p* < 0.001; *R* = 0.504, *p* < 0.001, respectively, *n* = 144). In 2017, *E. nuttallii* became the most abundant macrophyte species in Lake Müggelsee, while *S. pectinata* used to dominate the macrophyte community before 2017 (Figures 3A–C). Highest mean quantities of *E. nuttallii* were observed in depth zones 1–2 and 2–4 m (Figures 3B,C). In 2018, mean *E. nuttallii* quantities significantly declined as compared to 2017 (Figures 3A–C, Wilcoxon test, *p* = 0.05). Mean quantities of most native submerged macrophytes increased, in particular that of *N. major* in depth zones 0–1 and 1–2 m, of *C. demersum* in depth zones 1–2 and 2–4 m and of *F. antipyretica* in depth zone 2–4 m (Figures 3A–C). Mean maximum colonization depth (MCD) of macrophytes at eight transects increased from 0.9 ± 0.2 m in 1999 to 3.6 ± 0.3 m in 2018 (Figure 4A). Quantities of both, *E. nuttallii* and native macrophytes in the depth zone 2–4 m, were positively correlated with MCD (Spearman correlations, *R* = 0.463, *p* = 0.001; *R* = 0.441, *p* = 0.002, respectively, *n* = 48).

### Dreissenid Mussels

In 2017, dreissenid mussels covered about one third of the sediment surface of Lake Müggelsee. Grab samples taken in 2017 were composed of 97.3% *D. r. bugensis* and 2.7% *D. polymorpha*. A larger proportion of *D. polymorpha* (11.0% of all dreissenids) was only found in nearshore samples taken from rock fill and sand with high contents of stones. Maximum colonization depth was on average 5.3 m (Figure 1C). Population densities of dreissenids varied between 1,600 and 46,000 mussels m^−2^, with a median of 12,800 mussels^−2^. Weighted by the share of coverage classes, whole-lake density was 3,600 dreissenid mussels m^−2^ (Table 1). The effect of lake-sub-area on dreissenid mussel densities was significant (*F*_5,34_ = 4.56, *p* = 0.003). Two main sub-areas were considered by merging (1) N, NE, and E sub-areas into one (~42% of whole lake-area) with a median density of ~10,000 mussels m^−2^ (abundance ~8.6 × 10^9^) and (2) SE/S, SW/W, and NW sub-areas (58% of whole lake-area) with median densities of ~20,000 mussels m^−2^ (abundance ~19.6 × 10^9^). The total abundance of *D. r. bugensis* of ~28.2 × 10^9^ resulted in the clearance of filtered lake water about 1.9 times per day.

**Table 1:** Comparison of population densities of quagga mussels (*Dreissena r. bugensis*) in different lakes.

Lake	Investigated depths (m)	Mean quagga mussel density (n m^−2^)	Reference
Szczecin Lagoon (PL)	3.8–8.5	4,000 ± 355	Woźniczka et al., 2016
Lake Mead (USA)	2–112	747 ± 398	Wittmann et al., 2010
Lake Müggelsee (DE)	0.7–5.6	3,600 ± 1,200	This study
Lake Eem (NL)	2.1	2,300	Noordhuis et al., 2016
Lake Simcoe (CA)	2–20 >20	334 ± 65 39 ± 21	Ginn et al., 2018
Lake Ontario (CA)	5–20	9,400 ± 7,200	Wilson et al., 2006
Lake Huron (USA)	46–73	72–811	French et al., 2009
Lake Michigan (USA)	20–45	6,900 ± 4,500	Nalepa 2010
Lake Erie (USA)	0–>24	380 ± 40	Karatayev et al., 2014

**Figure 5:**
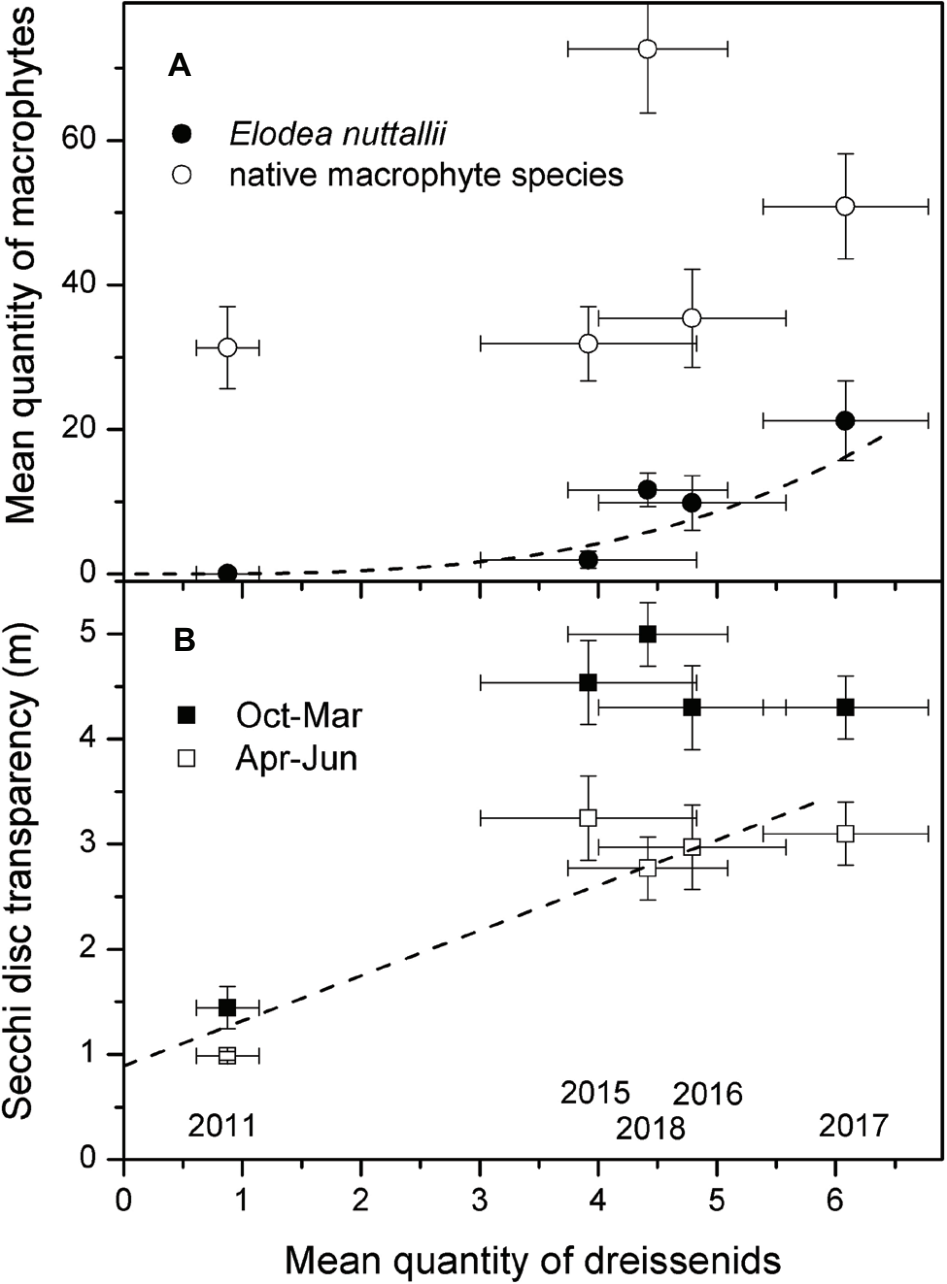
Relationships between the yearly mean quantities of dreissenid mussels and *E. nuttallii* or native macrophytes for the period 2011–2018 (*n* = 5) **(A)** and between yearly mean quantities of dreissenid mussels and mean Secchi disc transparency in winter (October to March) and spring (April to June) **(B)**. Lines represent significant regressions, years are given in **(B)**.

In 2011, dreissenid mussels comprised only *D. polymorpha* and 50% of the investigated 24 locations (three depth zones at each of the eight transects) did not have any dreissenid mussels. Abundance class 3 did not occur and abundance class 2 was only measured in depth zone 2–4 m at three transects. From 2015 (no data are available for the period 2012–14) onwards, much higher mean quantities were observed in all depth zones and highest values were reached in the depth zone 2–4 m (Figure 3D). In 2018, mean dreissenid quantities dropped as compared to 2017 (Figure 3D, Wilcoxon test, *p* = 0.05). When separately testing data of each year, a significant positive correlation was found between quantities of dreissenids and *E. nuttallii* in 2016 (Spearman correlation, *R* = 0.47, *p* = 0.02, *n* = 24), while quantities of native macrophytes did not show significant correlations with quantities of dreissenids. Using yearly means of all 24 locations, a significant correlation (non-linear regression: *y* = 0.06*x*^3.2^, *R*^2^ = 0.96, *p* = 0.004) was found between quantities of dreissenids and *E. nuttallii* (Figure 5A).

### Lake Water Quality

Secchi disk transparency in Lake Müggelsee showed low interannual variability and were only slightly higher in winter than in summer between 1999 and 2010. A significant increase in winter Secchi disk transparency was observed between 2010 and 2012, the period when *E. nuttallii* and *D. r. bugensis* colonized the lake. Mean summer Secchi disk transparency only increased significantly between 2013 and 2014 (Figure 4B). A significant linear regression was found between yearly mean quantities of dreissenids and mean Secchi disk transparency in April–June for the period 2011–18 (Figure 5B, linear regression: *y* = 0.43*x* + 0.89, *R*^2^ = 0.8, *p* = 0.04). Concentrations of Chl *a* (Figure 4C) and TP (Figure 4D) decreased in parallel with increased Secchi disk transparency (Figure 4B).

## Discussion

Our results provide empirical evidence for IMH during the initial establishment of two important aquatic invaders and suggest a subsequent change of the invader-invader-interaction into competition for space. We observed a significant correlation between quantities of dreissenids and *E. nuttallii* at the site level in 2016 and a significant non-linear regression between yearly means (2011–2018) of quantities of dreissenids and *E. nuttallii* at the whole-lake level, indicating a mutual facilitation between the establishment of quagga mussels (97% of dreissenids in 2017) and the waterweed in a temperate freshwater lake during the first 4–6 years of their invasion. Mussel filtration induced an increase in water clarity, supporting macrophyte colonization in deeper littoral zones, while macrophytes provided substrate for the attachment of young mussels and produced oxygen which may help to prevent hypoxia during summer months. We propose that the invasive *E. nuttallii* was able to rapidly and efficiently make use of the newly available habitat in deeper littoral areas once light availability increased compared to native macrophytes. This is due to its ability to spread by fragments, its rapid growth rates, survival into winter months, and compensation of losses by herbivory through branching. This can give *E. nuttallii* a temporal advantage over native macrophytes. About 5 years after starting the invasion, *E. nuttallii* dominated the submerged macrophytes, covering 25% and quagga mussels colonizing 33% of the lake’s sediment surface. In 2018, quantities of both dreissenids and *E. nuttallii* decreased, suggesting a competition for space in the lake which could be a reason for the boom-bust dynamics the latter species is known for. The spread of native macrophytes can re-inforce this process.

### Invasion of Waterweed and Quagga Mussels and Mutual Facilitation

Nuttall’s waterweed invaded Lake Müggelsee at some point between 2006, when it was not present, and 2011, when it was detected for the first time at two transects at the southeastern shore. This location suggests that *E. nuttallii* entered the lake *via* River Spree. The establishment in the lake took about 5 years until 2016 and 2017, when *E. nuttallii* was found at 6 and 8 out of 8 transects, respectively. Native macrophyte quantities increased at a slower pace, which confirms findings of Kelly et al. (2015) in Irish lakes, but contrasts laboratory experiments that show *E. nuttallii* can outcompete other submerged species (Barrat-Segretain, 2005). Despite a parallel increase in native macrophyte abundance, *E. nuttallii* was initially faster in colonizing the new habitat in deeper littoral areas after increased light availability than native macrophytes. We assume that this was due to a combination of traits such as rapid spread by fragments (Hussner, 2012), rapid growth rates, being wintergreen and growth at low temperature (Kunii, 1981) and compensating biomass losses by herbivory through branching (He et al., 2019). In addition, *E. nuttallii* can take up phosphorus (P) *via* shoots and roots (Angelstein and Schubert, 2008) and thus can directly make use of the P translocated by quagga mussels from pelagic to littoral areas (“benthic shunt”, Hecky et al., 2004). However, P was not a limiting factor for macrophyte growth in Müggelsee, as this would require much lower P concentrations in the water (Gross, 2009).

*E. nuttallii* invaded the lake when Secchi disk transparency was still rather low in summer and winter, while maximum colonization depths of macrophytes were already deeper than 3 m, but total macrophyte quantities in the depth zone 2–4 m were still lower than in shallower parts of the lake. From winter 2013/14 onwards, winter water clarity suddenly was about twice higher than before and from 2014 onwards, water clarity was also higher in summer. Quagga mussel invasion (see below) is assumed to be responsible for this effect. *E. nuttallii* has been observed to grow even at 4°C, shoot elongation starts in spring at about 10°C (Kunii, 1981) and green shoots have been found washed ashore at Lake Müggelsee in December 2017 (S.H., personal observation). Thus, it could probably take most advantage of the higher light availability in winter and spring and increase its abundance into deeper water before most other competing native species also spread into this depth zone. The native species *F. antipyretica* is also wintergreen (Maberly, 1985) and its strong increase in quantity in the depth zone 2–4 m suggests that this species also gained from higher water clarity in winter. However, due to its lower growth rate as compared to *Elodea* (Sand-Jensen and Madsen, 1991), the invasive *E. nuttallii* at least temporarily dominated the submerged vegetation of Lake Müggelsee in 2017.

Existing knowledge on mechanisms of invasions of aquatic plant communities is still limited (Fleming and Dibble, 2015). However, several examples also suggest a link between the spread of invasive macrophytes and *D. polymorpha* induced turbidity reductions in lakes (Skubinna et al., 1995; MacIsaac, 1996; Zhu et al., 2006). Because quagga mussels can colonize all regions of a lake, and form larger populations, they may filter larger water volumes and may thus have even greater effects on macrophyte abundance than *D. polymorpha*, which are restricted to shallower portions of lakes (Karatayev et al., 2015). In the Dutch Lake Eem, the establishment of quagga mussels was also paralleled by an increased macrophyte abundance, but only by native species (Noordhuis et al., 2016).

Quagga mussels in Lake Müggelsee constituted 97% of the total *Dreissena* population in 2017, indicating that their invasion started around 2012 based on a spread model for *D. r. bugensis* in Western Europe by Heiler et al. (2013). The lake has been invaded by the congener *D. polymorpha* decades earlier. This species, however, was limited to littoral areas with hard substrates (Karatayev et al., 2015). In contrast, *D. r. bugensis* can also colonize soft sediments (Karatayev et al., 2015) and thus could reach a mean maximum colonization depth of 5.3 m and a total coverage of about a third of Lake Müggelsee’s sediment in 2017. Quagga mussel densities in Lake Müggelsee were high in 2017, but similarly high values have also been found in other lakes (Table 1). Filtration capacities in Müggelsee were comparable to those found in Dutch shallow lakes (Noordhuis et al., 2016) and Lake Erie (Vanderploeg et al., 2002).

Although not directly measured, *E. nuttallii* were supposed to have supported quagga mussel invasion by provision of surface for attachment. A number of studies indicate that macrophytes provide a suitable substrate for dreissenid mussel attachment. Körner et al. (2002) found *D. polymorpha* being the most abundant invertebrates on submerged macrophytes in Lake Müggelsee. Musko and Bako (2005) reported that *D. polymorpha* represented 2–85% of all animals on submerged macrophytes and their density ranged between 9 and 2,000 individuals g^−1^ macrophyte dry mass. Although studies on quagga mussel abundance on macrophytes are lacking, we assume that macrophytes support zebra and quagga mussel attachment in a similar way. Diggins et al. (2004) reported submerged macrophytes as a refuge for zebra mussels during quagga mussel invasion in the North American Great lakes.

*E. nuttallii* (together with the native macrophytes) may also have supported quagga mussels by oxygen production. Low oxygen concentrations (De Ventura et al., 2016) limit the survival of dreissenids and regular annual hypoxia (oxygen concentrations below 2 mg L^−1^) excluded dreissenids (Karatayev et al., 2018a). Assuming an average increase in *E. nuttallii* biomass in June by about 100 g dry weight m^−2^ in dense stands (Cook and Urmi-König, 1985) and a carbon content of 33% dry weight (Velthuis et al., 2017), the additional oxygen production amounts to about 20 mg L^−1^ in a 4 m water column in 30 days. In comparison, phytoplankton would produce about 4 mg O_2_ L^−1^ (means of net production in 2011–2017, Köhler, unpublished data). Available studies show that macrophyte biomass can be the most influential environmental factor on the fluctuation of dissolved oxygen concentrations in the bottom water of lakes (Haga et al., 2006; Vilas et al., 2017). While aquatic plants are usually thought of as providing oxygen to aquatic environments, they can also engineer extremely low values (Caraco et al., 2006). Vilas et al. (2017) report on the development of night-time anoxic conditions close to the sediments when macrophytes occupied at least 50% of the water column and induced stratification.

### Competition for Space

Karatayev et al. (2015) suggested that quagga mussels provide additional space and food for many invertebrates in the littoral zone, and thus have overall positive impacts on the benthic community by increasing diversity, density, and biomass of invertebrates. In the profundal zone, however, quagga mussels compete for space and food resources with most of native invertebrates decreasing their overall diversity, density, and biomass. The decline in quantities of dreissenids and waterweed in Müggelsee in 2018 suggests that there can be competition for space among quagga mussels and macrophytes also in the littoral zone. According to Nalepa (2010), *D. r. bugensis* usually has a more even distribution and rarely forms large druses on soft sediments of the profundal. In Lake Müggelsee, dense carpets of quagga mussels were observed between 2 and 4 m (Figure 1D). These mussel carpets are assumed to prevent an attachment of *E. nuttallii* shoots to the sediments by roots. *Vice versa*, dense stands of *E. nuttallii* (Figure 1B) or native macrophytes could prevent a successful establishment and survival of quagga mussels due to particle retention (Vermaat et al., 2000) and allelopathic inhibition of phytoplankton (Erhard and Gross, 2006) resulting in insufficient food availability for quagga mussels. Indeed, low phytoplankton abundances (Figure 4C) might have affected the growth, recruitment and survival of quagga mussels in Müggelsee. Declining dreissenid recruitment and growth has been found following declines in food availability in the hypolimnion of Lake Erie (Karatayev et al., 2018b), so it would be worth following size-frequency distributions in the future. Higgins (2014), however, did not find evidence for diminished effects of dreissenids on ecosystems within two decades after their establishment in US waters.

We conclude that our observational study indicates a mutual facilitation between *E. nuttallii* and quagga mussels during the first years of their invasion, which subsequently turns into a competition for space. Observational approaches are re-emerging in ecology and have demonstrated their capability in testing hypotheses by correlating variables, comparing observed patterns to output from existing models and exploiting natural experiments (Sagarin and Pauchard, 2010). Ideally, they would be combined with experimental manipulations to isolate fine-scale ecological mechanisms. In our study, a full understanding of the interactions between dense dreissenid populations and macrophyte stands in lakes requires further detailed analyses, in particular on fluxes and mass balances of dissolved inorganic carbon and oxygen. For *E. nuttallii*, several other potential invasion mechanisms have been suggested, including enemy release, novel weapons/allelopathy, phenotypic plasticity, fluctuating resources, and opportunity windows (Fleming and Dibble, 2015 and references therein). In the case of Lake Müggelsee, none of these seems to be more likely than invasional meltdown.

## Author Contributions

SH conceived the presented idea and wrote the manuscript together with BW. BW conducted the dreissenid mapping in 2017 and KvdW the macrophyte and dreissenid mapping. JK provided lake and phytoplankton data. EF and MM performed molecular analyses. AK and MG provided background information on the lake and supported statistical analyses. All authors contributed to discussions and the writing of different parts of the text.

### Conflict of Interest Statement

KvdW is affiliated to the company lanaplan GbR. All other authors declare no competing interests.

## Footnotes

^1^https://blast.ncbi.nlm.nih.gov/Blast.cgi
